# Supplementary material for: Clinical laboratory hematology reference values among infants aged 1month to 17 months in Kombewa Sub-County, Kisumu: A cross sectional study of rural population in Western Kenya
Source: PLoS One. 2021 Mar 17;16(3):e0244786. doi: 10.1371/journal.pone.0244786 (PMC7968642; doi:10.1371/journal.pone.0244786)
Supplement: S3 File — (DOC) [file pone.0244786.s004.doc]

# 10Aug2016

# MEMORANDUM THRU Director, Walter Reed Project Kisumu, US Army Medical Research Directorate – Kenya, Unit 8900, Box 330, DPO AE 09831-0330

**FOR** Commander, Walter Reed Army Institute of Research (ATTN: Division of Human Subjects Protection), 503 Robert Grant Avenue, Silver Spring, MD 20910

S**UBJECT:** WRAIR#2325 Waiver of Informed Consent for Minimal Risk Research Protocol,

**PROTOCOL TITLE**: Hematology Reference Ranges in Healthy Children under 5 years in Kombewa Sub-County, Western Kenya

The above mentioned protocol has been determined as a minimal risk study. It is a study that will be using data extracted from a previously conducted Mal-055 GSK vaccine sponsored study. Mal-055 study (WRAIR 1547) was a phase III, double blind (observer-blind), randomized, controlled multi-center study to evaluate, in infants and children, the efficacy of the RTS,S/AS01E candidate vaccine against malaria disease caused by P. falciparum infection, across diverse malaria transmission settings in Africa. The study population was both male and female infants and children aged 6-12 weeks and 5-17 months of age at first vaccination if eligible according to inclusion and exclusion criteria.

The data for WRAIR# 2325 will be extracted from the Mal-055 database in 2 age brackets; at least 286 children from upper age group (5-17months old at screening) and at least 245 children from lower age group (6-12weeks at screening), will be randomly selected. The data extracted will be completely anonymized.

Studies involving human subjects normally would require informed consent to be administered to study subjects before participating in the study. The subjects that were enrolled under the major vaccine study had their parents/guardian provide written informed consent for participation in the main study. It is not be feasible at this point in time to go back to the field to get informed consent signed by the participants for the primary study for use of data for this study, given the time that has elapsed since the last subject was seen at the clinic. In addition, the informed consent obtained during the phase III Mal-055 trial stated that the subjects understood and granted access of data to authorized persons described in the information sheet. It stated that, ‘’*The information and test results that are collected may also be reviewed by representatives of the Kenya Medical Research Institute, WRAIR DHSP (Department of Human Subject Protection) the U.S. Army Medical Research and Materiel Command (USAMRMC), the US Food and Drug Administration and other regulatory authorities. Test results and other health information about your child will also be shared with the other research centers who are working with us on this*

*study’’*. A copy of the informed consent form is attached for reference

Given that I was part of the study laboratory team during the Mal-055 trial, and the study participants’ parents/legally accepted representatives consented to grant access to the test results and other health information about their children to research centers working with the study, I would like to request for a waiver of consent for this study. In addition, the research presents no more than minimal risk to subjects, involves the use of already anonymized data and involves no procedures for which written consent would normally be required, I would like to request for a waiver of consent to enable me carry out the data analysis for this project.

.

Point of contact: Jew Ochola, phone: +254 728 915 545 or email [jew.ochola@usamru-k.org](mailto:jew.ochola@usamru-k.org)

Sincerely,

Jew Ochola, BSC

USAMRU-K

[Jew.ochola@usamru-k.org](mailto:Jew.ochola@usamru-k.org)

+254 728 915 545
