# Supplementary material for: Clinical laboratory hematology reference values among infants aged 1month to 17 months in Kombewa Sub-County, Kisumu: A cross sectional study of rural population in Western Kenya
Source: PLoS One. 2021 Mar 17;16(3):e0244786. doi: 10.1371/journal.pone.0244786 (PMC7968642; doi:10.1371/journal.pone.0244786)
Supplement: S4 File — (PDF) [file pone.0244786.s005.pdf]

DEPARTMENT OF THE ARMY
WALTER REED ARMY INSTITUTE OF RESEARCH
503 ROBERT GRANT AVENUE
SILVER SPRING, MD 20910-7500

REPLY TO
ATTENTION OF

MCMR-UWZ-C 15 May 2017

MEMORANDUM FOR Jew Ochola, BSc, Kenya Medical Research Institute (KEMRI)/Walter Reed Project (WRP), P.O. Box 54 – 40100, Kisumu, Kenya

SUBJECT: Commander Approval Authorization of the Minimal Risk Human Subjects Research Protocol, **WRAIR #2325**

1. The protocol, **WRAIR #2325**, entitled "Hematology Reference Ranges In Healthy Children Under 5 Years in Kombewa Sub-county, Western Kenya," (Version 1.6, dated 7 February 2017), and supporting documentation have been submitted and reviewed in accordance with applicable Walter Reed Army Institute of Research (WRAIR) and Federal policies, procedures, and guidance.

2. The WRAIR Institutional Review Board (IRB) Chair approved protocol Version 1.5, dated 10 October 2016 via expedited review procedures on 8 November 2016. The IRB Chair approved Update #1 to the protocol (Version 1.6, dated 7 February 2017) via expedited review procedures on 6 April 2017. (See enclosures)

3. The following human subjects protection related documents pending at the time of the IRB approval have been received by the WRAIR Human Subjects Protection Branch (HSPB):

   a. KEMRI Scientific and Ethics Review Unit (SERU) approval of protocol Version 1.6, dated 7 February 2017, (received: 24 March 2017); and

   b. U. S. Army Medical Research and Materiel Command (USAMRMC), Office of Research Protections (ORP), Human Research Protection Office (HRPO) approval, dated 12 May 2017, (received: 12 May 2017).

4. As a reminder, the study expiration date is **8 November 2017**. The Principal Investigator (PI) is responsible for submitting a continuing review report to the WRAIR HSPB in time for the report to be reviewed and approved by the KEMRI SERU and the WRAIR IRB prior to the respective expiration dates in order for work to continue without interruption. A study closeout report or request for extension must be submitted to the WRAIR HSPB not later than five (5) years from the initial date of approval (**i.e. 8 November 2021**). No changes, amendments, or addenda may be made to the protocol without prior review and approval by the KEMRI SERU and the WRAIR IRB, as well as the USAMRMC ORP HRPO, as applicable.

5. The PI has the responsibility to obtain all business agreements prior to initiation of any work with partners/collaborators or contracted services. This includes any transfer of data or specimens. Failure to obtain business agreements prior to initiation could result in sanctions or disciplinary actions for both the Detachment Director and the PI. The IRB and HSPB will review business agreements as part of monitoring visits to ensure they were obtained as required and report to the WRAIR Commander as to adherence to this requirement.

MCMR-UWZ-C
SUBJECT: Commander Approval Authorization of the Minimal Risk Human Subjects Research Protocol, **WRAIR #2325**

6. As there are no outstanding human subjects protections issues, approval authorization is granted to this study (Version 1.6, dated 7 February 2017).

7. The point of contact (POC) for this action is Maya Foster-Brown, M.S., at (301) 319-9974 or maya.e.foster-brown.civ@mail.mil.

Deborah L. Whitmer
DEBORAH L. WHITMER
COL, VC
Commanding

Encl.
WRAIR IRB Approval Memoranda,
8 November 2016 and 6 April 2017

CF:
Michael Zapor, COL, MC
Douglas Shaffer, M.D.
Victor Melendez, LTC, MS
Kisumu Regulatory Affairs
Lucas Otieno, MBChB, MSc
Matthew Brown, MAJ, MS
Stacey Gondi
Margaret Odongo
Jody Ference, M.S., CIP, CCRA, CIM
MCMR-RP
