# Supplementary material for: Clinical laboratory hematology reference values among infants aged 1month to 17 months in Kombewa Sub-County, Kisumu: A cross sectional study of rural population in Western Kenya
Source: PLoS One. 2021 Mar 17;16(3):e0244786. doi: 10.1371/journal.pone.0244786 (PMC7968642; doi:10.1371/journal.pone.0244786)
Supplement: S5 File — (PDF) [file pone.0244786.s006.pdf]

MCMR-UWZ-C 6 April 2017

MEMORANDUM FOR Director, Human Subjects Protection Branch (HSPB), Walter Reed Army Institute of Research (WRAIR), 503 Robert Grant Ave., Silver Spring, MD 20910-7500

SUBJECT: Approval of Update #1 to the Minimal Risk Human Subjects Research Protocol **WRAIR #2325**

1. I approve the update #1 to protocol, **WRAIR #2325**, entitled "Hematology Reference Ranges in Healthy Children under 5 Years in Kombewa Sub-county, Western Kenya," (Version 1.6, dated 7 February 2017), submitted by Jew Ochola, BSC, Kenya Medical Research Institute (KEMRI)-Walter Reed Project, Kisumu, Kenya.

2. The update #1 includes modifications to the protocol concerning the option to use the entire database, if necessary, to increase power, instead of the subset of 531 subjects.

3. The WRAIR Scientific Review Committee (SRC) approved the protocol (Version 1.2, dated 22 July 2016) on 18 August 2016. Changes from Version 1.2 and 1.6 were minor and did not impact the science, so additional scientific review was not required.

4. Update #1 to the protocol is eligible for expedited review procedures in accordance with 32 CFR 219.110(b)(2) as minor changes to previously approved research. Data used in the study were previously collected under protocol WRAIR #1547.

5. KEMRI Scientific and Ethical Review Unit (SERU) granted initial approval of the protocol (Version 1.6, dated 7 February 2017) on 6 March 2017 with an expiration date of 5 March 2018.

6. This study continues to be supported by the Henry Jackson Foundation.

7. U.S. Army Medical Research and Materiel Command (USAMRMC) Office of Research Protections (ORP), Human Research Protections Office (HRPO) approval will need to be received prior to issuance of the Commander Approval Authorization

8. The following document is included as part of this approval: Protocol (Version 1.6, dated 7 February 2017).

9. The expiration date of this study at WRAIR is **8 November 2017**. The PI is responsible for submitting a continuing review report in time for the report to be reviewed and accepted/approved by the KEMRI SERU and WRAIR IRB prior to the respective expiration dates to avoid an interruption in work. A study closeout report or a request for an extension must be submitted to the WRAIR HSPB no later than **8 November 2021**. No changes, amendments, or addenda may be made to the protocol without prior review and approval by the WRAIR IRB, KEMRI SERU, and USAMRMC ORP HRPO, as applicable.

10. The point of contact for this action is Lara D'Agaro, at 301-319-9088 or lara.m.dagaro.ctr@mail.mil.

JEFFREY R. LIVEZEY
LTC, MC
Interim Chair, Institutional Review Board
Walter Reed Army Institute of Research

CF:
Douglas Shaffer, M.D.
Victor Melendez, LTC, MS
Jew Ochola, BSC
Stacey Gondi
Margaret Odongo
Kisumu Regulatory Affairs
MCMR-RP
