# Supplementary material for: Clinical laboratory hematology reference values among infants aged 1month to 17 months in Kombewa Sub-County, Kisumu: A cross sectional study of rural population in Western Kenya
Source: PLoS One. 2021 Mar 17;16(3):e0244786. doi: 10.1371/journal.pone.0244786 (PMC7968642; doi:10.1371/journal.pone.0244786)
Supplement: S2 File — (PDF) [file pone.0244786.s003.pdf]

GlaxoSmithKline
Vaccines

Protecting · Human · Kind

GlaxoSmithKline Biologicals SA
Siège Social : Rue de l'Institut, 89
B-1330 Rixensart
Belgique
Siège d'Exploitation
Avenue Pascal 2/6
B-1300 Wavre
Belgique
Tel: +32 (0) 10 85 31 11

January 22nd, 2015

Dr. Walter Otieno
KEMRI
Kombewa Clinical Research Centre
Off Kisumu Bondo Road
Opposite Kisumu West District Hospital
Kisumu, Kenya

Re: Ancillary Study Proposal to Mal-055

Dear Dr. Walter Otieno,

This letter is to inform you that the Ancillary Study Review Committee (ASRC) for the Malaria-055 study has reviewed and approves your research study titled "Population based haematological reference value for under 5 years children."

1. The ASRC agrees with the proposal;
2. The ASRC would like to encourage you to extend this analysis to other sites;
3. The ASRC would like to receive the analysis plan for information.

To keep our record updated you will be required to submit bi-yearly status reports, a final protocol, IRB approvals, if relevant any draft manuscripts or resulting study reports, when they are complete.

If you have any questions or need further assistance, please contact me (didier.h.lapierre@gsk.com) or my assistant Françoise Stevens (francoise.a.stevens@gsk.com, Tel +32 10 85 57 50).

We are very interested in your efforts and ask that you please do keep us informed of your progress.

Sincerely,

Didier Lapierre, MD
Chair, on behalf of
Ancillary Studies Review Committee
