# Supplementary material for: Clinical laboratory hematology reference values among infants aged 1month to 17 months in Kombewa Sub-County, Kisumu: A cross sectional study of rural population in Western Kenya
Source: PLoS One. 2021 Mar 17;16(3):e0244786. doi: 10.1371/journal.pone.0244786 (PMC7968642; doi:10.1371/journal.pone.0244786)
Supplement: Attachment — Submitted filename: Response letter 5 to the Reference Range Manuscript.docx [file pone.0244786.s007.docx]

**RESPONSE TO REVIEWERS- REBUTTAL LETTER-20Sep2019**

Reviewers' comments:

Reviewer's Responses to Questions

**Comments to the Author**

1. Is the manuscript technically sound, and do the data support the conclusions?

The manuscript must describe a technically sound piece of scientific research with data that supports the conclusions. Experiments must have been conducted rigorously, with appropriate controls, replication, and sample sizes. The conclusions must be drawn appropriately based on the data presented.

Reviewer #1: No

Reviewer #2: No

*RESPONSE: The manuscript has been completely revised and the authors are of the opinion that it is technically sound and its data supports conclusions. The sample size is acceptably high thus giving a high power.*

2. Has the statistical analysis been performed appropriately and rigorously?

*RESPONSE: A fresh round and completely new statistical analysis has been done which is more robust with a thorough confirmation. This has been described through the manuscript.*

Reviewer #1: Yes

Reviewer #2: No

3. Have the authors made all data underlying the findings in their manuscript fully available?

The [PLOS Data policy](http://www.plosone.org/static/policies.action#sharing) requires authors to make all data underlying the findings described in their manuscript fully available without restriction, with rare exception (please refer to the Data Availability Statement in the manuscript PDF file). The data should be provided as part of the manuscript or its supporting information, or deposited to a public repository. For example, in addition to summary statistics, the data points behind means, medians and variance measures should be available. If there are restrictions on publicly sharing data—e.g. participant privacy or use of data from a third party—those must be specified.

*The data has been attached as one of the supporting documents in the submission. Please attached file named ‘hematology reference range data’*

Reviewer #1: No

Reviewer #2: No

4. Is the manuscript presented in an intelligible fashion and written in standard English?

PLOS ONE does not copyedit accepted manuscripts, so the language in submitted articles must be clear, correct, and unambiguous. Any typographical or grammatical errors should be corrected at revision, so please note any specific errors here.

*RESPONSE-With another round of review of the manuscript, other typo and grammatical errors as have been noticed during this revision have now been corrected.*

Reviewer #1: No

Reviewer #2: No

5. Review Comments to the Author

Please use the space provided to explain your answers to the questions above. You may also include additional comments for the author, including concerns about dual publication, research ethics, or publication ethics. (Please upload your review as an attachment if it exceeds 20,000 characters)

Reviewer #1: This manuscript represents reference ranges for children under two years of age in a specific geographical location in Western Kenya. Whilst age and population specific reference ranges are extremely important for adequate clinical practice, this manuscript has major limitations that limit it from being published as is.

Major comments include:
1. English expression needs to be corrected throughout the manuscript.
2. Authors state that the ethics statement is "N/A". A clear and detailed statement is required for any study that has human participants or that uses human specimens or tissue.

The protocol, analysis and manuscript were undertaken following approval from both KEMRI SERU and WRIAR IRBs
3. The location of the data needs to be specified as per clear instructions provided by the journal. The statement "the underlying data is available" is not sufficient.

RESPONSE- *The minimal data set has been made available as one of the supporting information.* The title of the data document is ‘*hematology reference range data’. Relevant statement has also been added in the manuscript.*

4. Details of affiliations 1, 2, 3 need to be provided (e.g. city, country). The counties and countries for the affiliations have been specified in the manuscript. Line 7 and 8 of both tracked and clean manuscripts
5. The age median and range as well as gender proportions need to be provided for each age group. This has been edited and reviewed as required,

6. How is the age group 2 months to 6 months possible when the abstract stipulates that there was a gap in recruitment from 3 months (12 weeks) to 5 months?

RESPONSE-Please see the authors responses below:

*‘’The naming of age categories in the abstract is to show the age categories for the participants as they were in the primary study. The data for the reference range study was drawn from the subjects enrolled in the primary Mal-055 study, the infant and children age group for this study was made in line with the primary title. The primary study was titled- ‘A phase III, double blind (observer-blind), randomized, controlled multi-center study to evaluate, in infants and children, the efficacy of the RTS,S/AS01E candidate vaccine against malaria disease caused by P. falciparum infection, across diverse malaria transmission settings in Africa’.*

*The 6-12wks and 5-17months age categories of the primary study referred to enrollment periods. That is to mean that before enrolment at screening the participants could be some days/weeks before the enrollment period since there was a window period. So even the data set is available for the children who were able to be screened at 3 months, 4months as they were later enrolled To further clarify is that the screening data was used for those who were enrolled after meeting the inclusion criteria. For reference the Mal-055 protocol has been attached as supporting information for reference*

*The available age groups are fully included in the paper, unless apart from the ones that weren’t possible to be included due to the fact that they weren’t enrolled and not part of the primary study from which this study uses its data’’*

7. Methods - study area details are not required to the extent provided. Please focus on the population and recruitment only.

RESPONSE-This has been corrected
8. Sentences in lines 91 and 92 are not methods so please delete.

RESPONSE-This has been corrected
9. It is not clear how the 1509 patients were selected from the 1631 total. Who was excluded and why? The details of the excluded participants need to be specified.

*RESPONSE-the details of the excluded participants during data extraction have been included in the manuscript under exclusion criteria. Out of 1631 participants, 122 study participants were excluded from the analysis. These were participants whose data did not meet the study inclusion criteria for these analysis based on counter-verification of the medical history, physical examination and the hematology results. Figure 1 illustrated the basis of exclusion of these participants and this has also been described in the methods section which also includes how the 1509 were finally selected for the reference range analusis.*

10. BLOOD SAMPLES WERE COLLECTED "either through finger prick or venous draw". These two different processes of blood collection CAN NOT be treated as one. If all of the individuals are to remain in the study, the population details for finger prick versus venous collection need to be defined (e.g. how many participants, age, gender in each group) and what were the specific reference ranges for each of these sub populations. If the reference ranges are not signficantly different by these two methods of collection, then results need to be presented as one. ONLY UNDER THESE CIRCUMSTANCES.

RESPONSE-This has been corrected –Line 235 of the tracked manuscript and 138 of the clean manuscript
11. Results for all figures and tables need to be expanded in sentence form. As it stands, the results section is 10 sentences long. This is not appropriate for a study of this magnitude.
12. p-values as a comparison across age groups need to be presented. These are missing from the first part of table 1.

RESPONSE-p values have been included in comparison of the age groups
13. Comparison with other studies in table 3 is not useful unless appropriate p-values are presented to support the conclusions of whether there are or are not differences.

RESPONSE-In the first round of review of the manuscript, the authors were requested that they need to compare current results with other published articles which they need and so this hasn’t featured until in this review. Specifically the reviewer’s comment were as below

*Reviewer #1: 1. The aim of this study was to establish age-specific hematological reference values for “healthy” children who participated in malaria vaccine trials living in Kombewa, in rural Kenya. The paper is fairly well written with a good introduction and description of their work.
2. The authors’ main premise of carrying out the study is that reference intervals from elsewhere may not be appropriate in their setting. Nonetheless, they scantly compare with currently used intervals. Additionally, there are recently published intervals from within Kenya and East Africa, how do their findings compare with these? In line 240-244 they mention variation of their findings with data in Harriet Lane Handbook.*

*They needed to delve deeper in this and show how significant this variation is.*

Reviewer #2: The purpose of this manuscript was to provide targeted hemotological reference ranges for children under two years old in Kombewa Sub-county, Western Kenya. Although I agreed this is an important topic to pursue, I found some weaknesses of this study.

Here is a list of specific comments. Note: line and page numbering in reviews and comments is based on ruler applied in Editorial Manager-generated PDF.

1. Page 3, line 68: Did the sample selection process target the HDSS sub-county rural population? It was important to show the sample in this study was a good representative of the target population.

RESPONSE-Yes, this is well clarified in lines 115-116 of the clean manuscript and 164-165 of the tracked manuscript

2. Page 5, line 105: Were the healthy subjects part of the clinical trial, NCT00866619? Please clarify in the manuscript what the “healthy subjects” meant in relation of the “children” listed in the previous three bullet points.

RESPONSE-Yes, healthy subjects were part of the clinical trial. Further clarification to this given in the manuscript as follows;

- without acute or chronic, clinically significant pulmonary, cardiovascular, hepatobiliary, gastrointestinal, renal, neurological, mental or hematological functional abnormality or illness that required medical therapy, as determined by **medical history, physical examination** (blood pressure, weight, pulse, Z score and vital signs) or clinical assessment before being enrolled into the study.

*During the primary vaccine study, the children who were enrolled and vaccinated in the study had all their physical examination done. Thus this data set being drawn from the children enrolled in the primary study had normal physical examination. Besides this was confirmed during extraction and verified by the principal investigator before being used in the analysis*.

The terminology for ‘children’ also refer to the ‘participants’, the latter being retained in some cases being in the title. ‘Subjects’ have been replaced with ‘participants’.

3. Page 5, line 117: Was the major study the same as the primary study? If so, I suggest to describe the study consistently. If no, please distinguish the difference in the manuscript.

RESPONSE-This has been corrected,
4. Page 6, line 121: Were the “subjects” the same as the “children”?

Yes-the terminology ‘’subjects’’ referred to the children

5. Page 6, line 129: Again, I suggest to use one of “subjects”, “children”, and “participants” consistently throughout the manuscript.
RESPONSE-The terminology for ‘children’ also refer to the ‘participants’, the earlier being retained in some to be synonymous with the title. ‘Subjects’ have been replaced with ‘participants’.

6. Page 8, lines 181–182: I think you meant to state ‘to present the descriptive statistics non-parametrically such as median and 2.5th and 97.5th percentiles for continuous variables and frequencies for categorical variables’. In fact, there was no frequency presented in the manuscript.

RESPONSE-This has been edited

7. Page 8, lines 182–185: The Mann-Whitney U and Kruskal-Wallis tests were OK. However, did comparing the hematological values among groups have anything to do with establishing the hematology reference values?

RESPONSE-Yes, we needed to get a thorough understanding of the differences between the groups statistically if they exist without leaving anything to chance

8. Page 8, lines 186–187: The phrase “Vol 20 No 30 . . . as to avoid bias” was not easy to understand. How about ‘outliers were not eliminated in this study’?

RESPONSE-the statement has been reframed and edited

9. Page 9, line 194: The phrase “the Kombewa laboratory reference values consort diagram” did not read well and did not match what Fig 1 depicted.

RESPONSE-In the initial and first round of review of the manuscript, reviewer suggested we put a Kombewa laboratory reference values consort figure that would show the final numbers as were used for establishing the reference ranges following exclusion of others. Thus we developed this after that review and so it didn’t come as a comment on the subsequent review.

10. Page 9, line 196: I would suggest to have a table describing the characteristics of these 1509 healthy subjects. Especially, how could this sample of 1509 healthy subjects represent the population in Kombewa Sub-county in western Kenya?

Response-representation for the sample to the population is illustrated in the methods section and is more than the recommended sample size of 240 as recommended by CLSI guidelines

11. Page 9, line 202: I suggest to list all hematological analytes presented in Fig 2 rather than “some”.
RESPONSE-this has been corrected and all analytes have been included in the current tables
12. Page 9, line 203: Was there an algorithm “deriving” the reference ranges? Or, the reference ranges were simply summary statistics from the sample. If so, the term “derived” was misleading.

Response-The term derived has been replaced with established.

13. Page 14, lines 222–223: Why did Fig 3 not include all combination of age and gender groups for all hemotological analytes?

All combination of ages and analytes have been included in this revision

6. If you would like your identity to be revealed to the authors, please include your name here (optional).

Your name and review will not be published with the manuscript.

Reviewer #1: (No Response)

Reviewer #2: (No Response)

[NOTE: If reviewer comments were submitted as an attachment file, they will be attached to this email and accessible via the submission site. Please log into your account, locate the manuscript record, and check for the action link "View Attachments". If this link does not appear, there are no attachment files to be viewed.]

- - - - -
For journal use only: PONEDEC3
