# Supplementary material for: Clinical laboratory hematology reference values among infants aged 1month to 17 months in Kombewa Sub-County, Kisumu: A cross sectional study of rural population in Western Kenya
Source: PLoS One. 2021 Mar 17;16(3):e0244786. doi: 10.1371/journal.pone.0244786 (PMC7968642; doi:10.1371/journal.pone.0244786)
Supplement: Attachment — Submitted filename: Response to Querry-27July2020.docx [file pone.0244786.s008.docx]

**Response to Querry-27July2020**

PONE-D-19-28401R1

Clinical Laboratory Hematology Reference Values among Infants Aged 1month to 17months in Kombewa Sub-County, Kisumu: A cross sectional study of rural population in Western Kenya Mr Jew Ochola Ouma

Dear Mr Ouma,

Thank you for submitting your manuscript entitled "Clinical Laboratory Hematology Reference Values among Infants Aged 1month to 17months in Kombewa Sub-County, Kisumu: A cross sectional study of rural population in Western Kenya" to PLOS ONE. Your manuscript files have been checked in-house but before we can proceed we need you to address the following issues:

1. Further to previous requests we do need to ask you to address our requests with regards to S1 Table that you refer to on page 15 line 554. We do note that you have uploaded the file "Table 1-Supporting information.docx" however this appears to contain a duplicate fo your Table 2 that you have included in the manuscript on page 9.

Could you therefore please upload S1 Table, or if there is no separate S1 Table please remove the reference to it from your manuscript.

Response- We thank you for this correction. As there is no separate S1 table apart from the	table 2 included in the manuscript, the reference to it has been removed

Your manuscript has been returned to your account. Please log on to PLOS Editorial Manager at <https://www.editorialmanager.com/pone/> to access your manuscript.

Your manuscript can be found in the "Revisions Sent Back to the Author" link under the New Submissions menu. After you have made the changes requested above, please be sure to view and approve the revised PDF after rebuilding the PDF to complete the resubmission process.
